# Engineering mRNA‐LNP Medicines for the Ageing Brain: Opportunities and Challenges for Neurodegenerative Diseases

**DOI:** 10.1002/exp2.70223

**Published:** 2026-09-08

**Authors:** Abdel Ali Belaidi, Denzil L. Furtado, Francesca Alves, Rebecca M. Nisbet, Scott Ayton, Ashley I. Bush

**Affiliations:** ^1^ The Florey Institute of Neuroscience and Mental Health Parkville Victoria Australia; ^2^ The Florey Department of Neuroscience and Mental Health University of Melbourne Parkville Victoria Australia; ^3^ Zitra Medicines Pty Ltd, Carlton Victoria Australia

**Keywords:** Alzheimer's disease, blood–brain barrier, lipid nanoparticles, mRNA, neurodegenerative diseases

## Abstract

Messenger RNA (mRNA) therapeutics delivered by lipid nanoparticles (LNPs) have advanced from concept to clinic at unprecedented speed, yet their promise for neurodegenerative diseases remains largely untapped. Currently intractable age‐related proteinopathies such as Alzheimer's disease, Parkinson's disease, and amyotrophic lateral sclerosis demand new therapies that combine molecular precision with scalable manufacturing. This review surveys recent advances in engineering LNPs that traverse the blood–brain barrier (BBB), evade innate immune surveillance, and achieve cell‐selective expression in the ageing brain. We highlight emerging chemistries, including BBB‐shuttling ionizable lipids, peptide‐functionalized shells, and liver‐detargeted formulations that enable systemic or minimally invasive delivery to the central nervous system (CNS) in animal models. Although no CNS‐directed mRNA‐LNP has yet reached clinical trials, first‐in‐human studies for rare metabolic disorders demonstrate favourable safety, manufacturability, and durable protein replacement. Drawing lessons from COVID‐19 mRNA vaccines and ongoing liver‐targeted programmes, we outline a translational roadmap for brain applications. Major hurdles that are critically assessed include efficient endosomal escape in aged neurons, heterogeneity of the senescent BBB, chronic‐dose immunogenicity, and large‐scale synthesis of next‐generation lipids. Finally, we propose design rules and analytical standards to address outstanding knowledge gaps in expression durability and age‐related BBB alterations. Together, these insights chart a path for engineering mRNA‐LNP therapeutics capable of meeting the rising burden of neurodegenerative diseases in an ageing global population.

## Graphical abstract

This review highlights recent advances in engineering messenger RNA (mRNA)‐lipid nanoparticles (LNPs) to cross the ageing blood–brain barrier and target neurodegenerative diseases. It outlines design principles, delivery routes, and translational challenges, charting a roadmap towards clinical application of mRNA‐LNP therapeutics for neurodegenerative diseases.

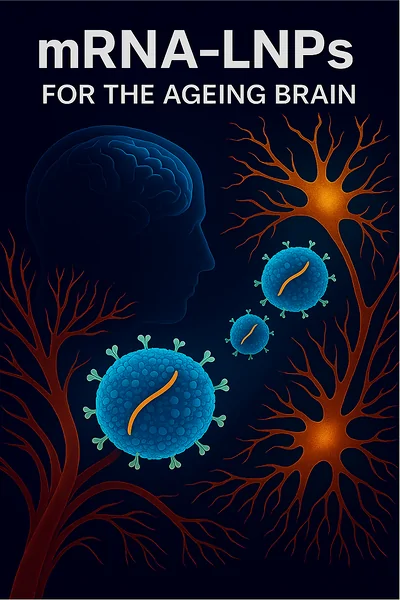

## 1 Introduction

Neurological disorders represent the largest contributor to global disability‐adjusted life years [1], with Alzheimer's disease (AD), Parkinson's disease (PD), and amyotrophic lateral sclerosis (ALS) accounting for a steadily increasing burden as populations age [2]. Existing small molecule and antibody therapies provide limited disease‐modifying benefit, and protein therapeutics face practical challenges related to high production costs and cold‐chain dependency. These realities underscore the need for scalable strategies capable of restoring or augmenting protein function directly within affected neural circuits [3].

Messenger RNA‐lipid nanoparticles (mRNA‐LNPs) technologies demonstrated unprecedented manufacturing speed and adaptability during the COVID‐19 pandemic [4, 5, 6], establishing a broadly applicable, cell‐free bioproduction platform. Unlike DNA‐based approaches, mRNA therapeutics do not require nuclear entry and are inherently non‐integrating, offering safety advantages for chronic conditions. Viral vectors such as adeno‐associated viruses have proven effective for DNA delivery and enabled the approval of the first gene therapy of the central nervous system (CNS), onasemnogene abeparvovec (also known as Zolgensma), for spinal muscular atrophy [7, 8, 9, 10, 11], but concerns about host immunity, insertional mutagenesis, and dose‐dependent liver toxicity continue to limit their broader deployment [12, 13, 14, 15, 16, 17]. These constraints have accelerated development of non‐viral delivery modalities, including lipid‐based and polymer‐based carriers, cell‐penetrating peptides (CPPs), and inorganic nanoparticles [11, 18, 19, 20]. Within the purview of non‐viral RNA delivery, and especially mRNA delivery, several different carrier systems have been studied, including LNPs [21, 22, 23, 24, 25], polyplexes [26], polymer‐LNPs [27, 28, 29], virus‐like particles [30], collagen matrices [31], CPPs [32], and N‐acetylgalactosamine conjugates in the case of siRNA delivery [33]. Of these, LNPs are by far the most well‐studied and best‐validated delivery platform to date [34, 35].

Despite substantial advances in systemic mRNA medicines, no LNP‐mediated mRNA therapy has yet been translated to the CNS, reflecting the combined challenges of the blood–brain barrier (BBB), neural innate‐immune surveillance, and the need for cell‐type‐specific expression [36]. At the same time, several categories of neurodegenerative diseases are conceptually well suited for mRNA‐based intervention, notably loss‐of‐function disorders where protein restoration is sufficient for therapeutic benefit (e.g., SMN1 deficiency in spinal muscular atrophy, enzyme deficiencies in leukodystrophies, or impaired antioxidant pathways in select ALS subtypes). More complex disorders such as AD and PD may also benefit from delivery of neuroprotective or modulatory proteins, including growth factors, lipid‐transport regulators, or protective receptor variants. These examples illustrate how emerging CNS‐directed LNP platforms may align with diverse therapeutic needs without attempting to catalogue disease‐specific mechanisms exhaustively.

This review therefore has four aims: (i) to survey chemical and nanotechnological strategies that facilitate LNP transit across the ageing brain; (ii) to distil insights from systemic mRNA‐LNP clinical programmes; (iii) to identify biological, immunological and manufacturing constraints unique to an ageing CNS; and (iv) to outline a translational framework that could enable mRNA‐LNP neurotherapeutics to reach first‐in‐human trials in the coming decade.

## 2 mRNA‐LNP Fundamentals

### 2.1 MRNA Chemistry

Therapeutic mRNA has evolved beyond a bare open‐reading frame capped with a generic 7‐methyl‐guanosine (m^7^G) and terminated by a poly(A) tail; contemporary transcripts weave together four interlocking layers of chemical and structural optimization that collectively balance translational output, innate‐immune invisibility and large‐scale manufacturability [37, 38, 39]. First, 5′‐cap engineering has progressed from the clinical mainstay Cap‐1 (m^7^GpppN1m) to anti‐reverse cap analogues and trinucleotide CleanCap reagents that elevate co‐transcriptional capping efficiency above 95%, trimming away costly enzymatic polishing, while hydrophobic photocleavable caps permit organic‐phase removal of abortive transcripts and “just‐in‐time” deprotection moments before LNP encapsulation [40, 41, 42]. Second, along the backbone, wholesale replacement of uridine with N1‐methyl‐pseudouridine, 5‐methoxy‐uridine, or 5‐methyl‐cytidine blunts Toll‐like receptors TLR3/7/8 and RIG‐I (Retinoic Acid‐Inducible Gene I) activation, and carefully placed phosphorothioate linkages at each terminus shield the strand from exonucleases without depressing translation [43, 44]. Third, sequence engineering now harnesses empirical screening and machine learning, exemplified by the Linear Design algorithm, to co‐optimize codon usage, GC content, secondary structure, and RNA‐binding motifs, boosting spike‐protein yield two‐ to four‐fold even as it shortens untranslated region (UTR) sequence length and lowers reagent consumption; the same framework can append tissue‐specific microRNA targets such as miR‐122 to serve as “off‐switches” that silence off‐target expression [45, 46].

Finally, next‐generation RNA architectures add another tier of control: circular mRNA (circRNA) produced via permuted introns or twister‐ribozyme autoligation resists 5′→3′ exonucleases and sustains translation for a week or more in vivo, whereas self‐amplifying RNA (saRNA) derived from alphavirus replicons reaches equivalent protein levels at one‐tenth the dose, albeit with longer ORFs that strain LNP loading limits [47, 48, 49]. However, early saRNA vaccine trials showed low efficacy due to high immunogenicity as compared to conventional mRNAs [50, 51]. This problem has been solved by the use of modified nucleotides in the RNA sequence, which conferred innate immune evasion and achieved robust long‐term protein expression [52]. A recent study investigating the therapeutic potential of circRNA for ocular neurodegenerative diseases like glaucoma showed that nerve growth factor (NGF)‐encoding circRNA in LNPs exhibits an exceptional capacity for prolonged protein expression and preserves retinal‐ganglion‐cell viability and axonal integrity, while significantly outperforming NGF protein therapy without detectable retinal toxicity [53]. Key structural determinants of mRNA performance are summarized in Table 1.

**TABLE 1:** Structural optimization strategies for mRNA therapeutics.

Feature	Design parameter	Typical variants	Functional impact	Limitations	Translational considerations
**5′ Cap structure**	Cap 0, Cap 1, ARCA, CleanCap	Cap 1 (m^7^GpppNm) standard; CleanCap highly efficient	Enhances translation initiation via eIF4E binding; reduces innate immune activation (Cap 1)	Cap 0 is more immunogenic; ARCA requires chemical synthesis; enzymatic capping adds complexity	CleanCap enables >90%–95% capping efficiency and is scalable for GMP manufacturing
**Poly(a) tail length**	Tail length (nt)	∼80–150 nt optimal	Improves mRNA stability and translation via PABP recruitment and mRNA circularization	Very short tails reduce translation; excessively long tails provide diminishing returns and may affect manufacturability	Defined poly(A) encoding improves batch consistency; enzymatic tailing introduces variability
**Codon optimization**	Codon usage bias; GC content	Moderate GC enrichment (∼50%–65%)	Enhances translational efficiency and mRNA stability	Excess GC can increase secondary structure and impair ribosome scanning; it may alter translation kinetics and protein folding	Requires balance between efficiency and biological fidelity; AI‐guided design is increasingly used
**RNA secondary structure**	5′ UTR, coding sequence folding	Minimized secondary structure near 5′ end	Improves ribosome accessibility and translation initiation	Over‐optimization may disrupt regulatory motifs	Requires computational modelling for optimal design
**Innate immune modulation**	Sequence composition; modified nucleosides	Use of modified bases (e.g., pseudouridine)	Reduces activation of TLRs and RIG‐I pathways; improves tolerability	May affect translation kinetics depending on context	Critical for repeated dosing and chronic therapies

Together, these innovations have stretched the practical payload window from less than one kilobase a decade ago to well over 10 kilobases today, opening the door to sophisticated cargos such as multi‐domain intrabodies, base editors, and polycistronic neurotrophic‐factor cocktails that hold promise for tackling neurodegenerative diseases.

### 2.2 LNP Architecture

**FIGURE 1:**
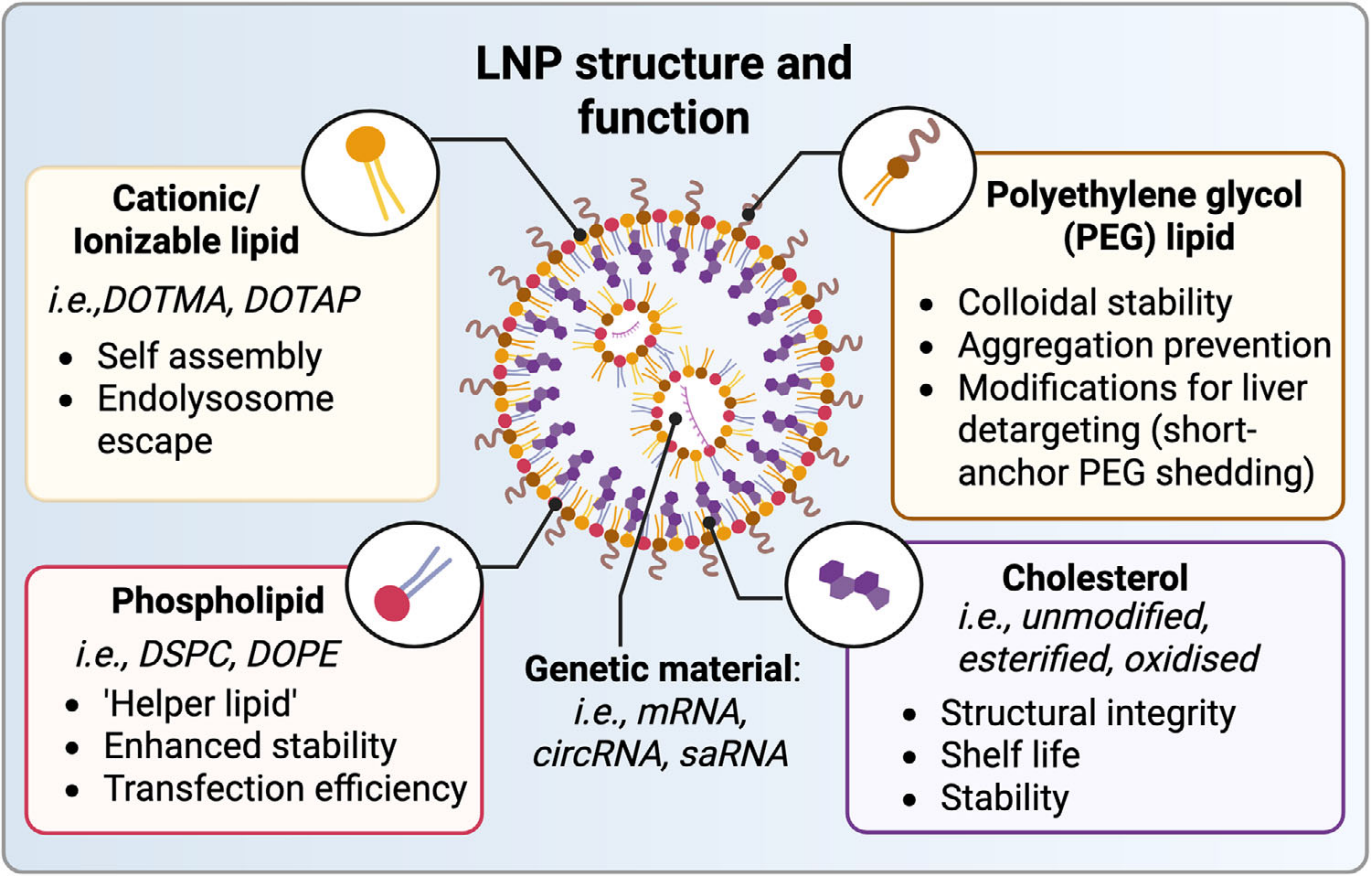
LNP structure and function. LNPs classically comprise four components: (1) a cationic or ionizable lipid (i.e., 1,2‐di‐O‐octadecenyl‐3‐trimethylammonium‐propane (DOTMA) and 1,2‐dioleoyl‐3‐trimethylammonium‐propane (DOTAP)), (2) a phospholipid (i.e., 1,2‐distearoyl‐sn‐glycero‐3‐phosphocholine (DSPC) and 1,2‐dioleoyl‐sn‐glycero‐3‐phosphoethanolamine (DOPE)), (3) cholesterol (which can be unmodified, esterified or oxidized), and (4) a polyethylene glycol (PEG) lipid. LNPs can encapsulate a range of nucleic acids and related cargo. Common types of genetic material used include messenger RNA (mRNA), circular mRNA (circRNA), and self‐amplifying RNA (saRNA).

LNPs classically comprise four components: (1) a cationic or ionizable lipid, (2) a phospholipid, (3) cholesterol, and (4) a polyethylene glycol (PEG) lipid (Figure 1), each of which is required in order to overcome the aforementioned challenges associated with in vivo delivery [34]. The standard percentage molar ratio of each lipid component is 50:10:38.5:1.5 (cationic/ionizable lipid:phospholipid:cholesterol:PEG lipid), although several variations of this have also been used [24]. The cationic or ionizable lipid serves to readily form complexes with negatively charged nucleic acids such as RNA, thus facilitating a highly efficient self‐assembly process (ionizable lipids are positively charged at low pH, which enables complexation when carried out in acidic buffer) [54]. Equally important, cationic and ionizable lipids induce endosomal escape once LNP is endocytosed into cells, thus allowing encapsulated nucleic acids to escape lysosomal degradation. Endosomal escape is thought to be induced by lipid‐mediated electrostatic interactions with the negatively charged endosomal bilayer, which subsequently results in membrane fusion and bilayer destabilization, followed by the release of therapeutic cargoes into the cytoplasm [55]. However, the efficiency of this process using existing materials is estimated to be extremely low, around 1%–2% [56]. Meanwhile, the phospholipid acts as a “helper” lipid, enhancing LNP fusogenicity and endosomal escape or increasing LNP stability, depending upon the choice of headgroup [55, 57]. Cholesterol contributes to the shelf life and in vivo stability of LNP formulations by interacting with phospholipids to fill in defects in the LNP membrane [57, 58]. Finally, the PEG lipid, which comprises a PEG polymer conjugated to a hydrophobic acyl chain, serves to increase colloidal stability, prevent LNP aggregation, and increase blood circulation time by reducing serum protein opsonization and reticuloendothelial clearance [57].

#### 2.2.1 Cationic/Ionizable Lipids

The first generation of liposomal and LNP formulations used cationic lipids such as 1,2‐di‐*O*‐octadecenyl‐3‐trimethylammonium‐propane (DOTMA) and 1,2‐dioleoyl‐3‐trimethylammonium‐propane (DOTAP) [59, 60]. However, these materials maintain their cationic charge in a pH‐independent manner, and studies have shown that they can exert toxic and immunomodulatory effects due to non‐specific interactions with serum proteins and toll‐like receptors located on macrophages and other sentinel cells [61, 62, 63, 64]. As such, subsequent efforts have revolved around the development of ionizable lipids: pH‐dependent lipids that maintain neutral charge at physiological pH and thus demonstrate lower general toxicity, but which take on a positive charge at low pH due to the protonation of free amines. This phenomenon is advantageous for the exploitation of endo‐lysosomal trafficking, since the pH of endosomes gradually drops from 6.8 to 4.5 as they traffic to the lysosomes, thus providing ionizable lipids with a brief window wherein to induce endosomal escape [65]. The structure of ionizable lipids typically includes an ionizable polar headgroup attached via a linker to one or more hydrocarbon chains. Structure‐activity relationship studies indicate that small variations in headgroup chemistry, linker biodegradability, and lipid tail branching can significantly influence both endosomal escape efficiency and tissue tropism, reinforcing the importance of iterative lipid design. Each of these individual moieties can be varied to determine the optimal parameters for transfection [66], but the key chemical property considered essential for effective delivery is the p*K*_a_ of the ionizable lipid when assembled into LNP form. The p*K*_a_ determines the surface charge of the LNP under different pH conditions and should ideally be between 6.0 and 6.5 to enable the optimal trade‐off between limited surface charge in circulation and positive surface charge in the endosomes [66].

The first clinical success with ionizable lipids occurred with DLin‐MC3‐DMA, a lipid containing a tertiary amine headgroup, ester linker, and dual unsaturated alkyl tails, which was used in an LNP formulation containing siRNA against transthyretin for the treatment of hereditary transthyretin amyloidosis [67]. Alnylam Pharmaceuticals shepherded the drug, called Patisiran, through clinical trials, and in 2018, it was approved for use in the United States and European Union under the brand name Onpattro [68]. Since then, several groups have launched separate efforts to optimize the structure of ionizable lipids for efficient delivery of even larger payloads such as mRNA. Moderna, for example, developed a biodegradable ionizable lipid with an ethanolamine headgroup and modified lipid tails containing ester linkages in place of di‐linoleic alkyl chains [24]. This lipid enabled more efficient delivery of mRNA in rats and cynomolgus monkeys when compared to DLin‐MC3‐DMA, while also improving the lipid clearance profile by virtue of the ester linkages, which are easily metabolized by esterases in vivo [24].

Although clearance profiles are often not prioritized in LNP development, an efficient lipid clearance profile is essential for preventing the build‐up of toxic LNP metabolites that can otherwise cause on‐ and off‐target toxicity in applications requiring repeat dosing [24]. Multiple strategies have been explored to improve biodegradability, including incorporating ester or disulphide linkers into the hydrophobic tails and altering headgroup chemistry to accelerate clearance [24, 69]. For example, Liu et al. reported in 2020 that attaching a tertiary amine with dual lipid tails to a phosphate‐containing single tail enhanced degradability and reduced hepatic retention [70]. The result was an ionizable phospholipid harbouring three lipid tails, which had the capacity to adopt a zwitterionic headgroup upon protonation of the amine in the low pH of the late endosome. This pH‐induced charge switching was shown to facilitate improved interactions with phospholipids in the endosomal membrane, resulting in increased bilayer destabilization and more efficient endosomal escape in vivo than DLin‐MC3‐DMA and other conventional lipids [70].

One of the drawbacks associated with many nanoparticles, including most LNP systems, is the fact that in vivo biodistribution tends to be heavily biased towards hepatic tissues. While this is beneficial for the treatment of primarily hepatic disorders such as methylmalonic acidaemia [21] and alpha 1‐antitrypsin deficiency [71], it is problematic for applications wherein extrahepatic tissues require efficient transduction [72]. Recent efforts have focused on integrating other materials into LNP formulations to alter tissue tropism patterns and enable more targeted transduction of desired organs.

For example, Cheng et al. showed that incorporating low amounts of a permanently cationic lipid (up to 30% as a fraction of total lipid content) into LNP formulations in addition to the ionizable lipid shifted the biodistribution profile from predominantly liver‐targeting in mice to predominantly spleen‐targeting, while increasing the fractional amount of cationic lipid beyond 30% resulted in a shift to predominantly lung‐targeting [73]. Meanwhile, incorporating even low amounts of various anionic lipids instead of cationic lipids resulted in almost exclusive delivery to the spleen. Furthermore, supplementing the standard LNP formulation with 20% extra ionizable cationic lipid (again, as a fraction of total lipid content) dramatically increased the hepatic transduction capacity of LNPs [73]. Separate efforts to transduce the spleen using LNPs with modified ionizable lipids and lower resultant p*K*a (∼5.7) have also been found to be effective [74]. In addition, Ramishetti et al. showed that LNPs formulated with ionizable lipids containing either hydrazine or hydroxylamine groups and conjugated with an anti‐integrin β_7_ monoclonal antibody were able to efficiently target CD4^+^ and CD8^+^ splenic T lymphocytes [75]. Degradable polymeric systems, particularly poly(β‐amino esters), have emerged as promising alternatives to classical cationic polymers due to their hydrolytically labile ester backbone, which enables improved biodegradability and reduced long‐term cytotoxicity. These systems have demonstrated efficient mRNA delivery and organ‐specific targeting in vivo, including mRNA delivery to pulmonary endothelial cells [27]. However, in contrast to ionizable lipid‐based LNPs, polymeric nanoparticles often require extensive optimization of polymer composition and formulation parameters, including PEG content and particle size, to achieve consistent efficacy. This structural and formulation complexity, combined with limited clinical validation, currently represents a key barrier to their translation compared to established LNP platforms.

#### 2.2.2 Phospholipids

While ionizable lipids have received the lion's share of engineering efforts, LNP‐containing phospholipids also play an important role in dictating carrier properties. Phosphatidylcholine‐based phospholipids, which comprise quaternary amines and are natural components of biological membranes, enhance the formation of lamellar phases and thus are useful in enhancing the stability of LNPs [57]. Saturated phosphatidylcholines such as 1,2‐distearoyl‐sn‐glycero‐3‐phosphocholine (DSPC) also have a high melting temperature (T_m_), thus further enhancing LNP stability relative to phospholipids with low T_m_ [76]. On the other hand, phosphatidylethanolamine‐based phospholipids such as 1,2‐dioleoyl‐sn‐glycero‐3‐phosphoethanolamine (DOPE), which contain protonated primary amines instead of quaternary amines, have been known to enhance the non‐lamellar hexagonal phase that is characteristic of the endosomal membrane's transitionary structure during LNP‐induced bilayer destabilization [77]. These differences between phospholipid types amount to a trade‐off between stability and transfection efficiency. While some groups prefer the enhanced capacity for endosomal escape that results from incorporating DOPE into LNPs [78], other groups favour the improved stability and encapsulation efficiency that is derived from DSPC [24]. Naturally, lipid tail length and degree of saturation also play important, albeit secondary, roles in determining the inherent chemical activity of each class of phospholipid [76].

#### 2.2.3 Cholesterol

Unmodified cholesterol is typically incorporated in LNP formulations with the purpose of providing structural integrity. However, recent studies have shown that modifying the structure of cholesterol can affect both the transduction efficiency and tissue‐targeting properties of LNPs [79, 80]. In a study of six different cholesterol variants, including unmodified cholesterol, two different cholesterol esters, and three different forms of oxidized cholesterol, Paunovska et al. found that LNPs formulated with cholesterol oleate delivered RNA constructs to hepatic endothelial cells with threefold greater efficiency than hepatocytes [80]. This was in contrast to LNPs formulated with unmodified cholesterol, which were unable to target endothelial cells as efficiently. Separately, Patel et al. showed that mRNA‐containing LNPs formulated with the cholesterol analogue ß‐sitosterol were able to transfect various immortalized and primary cell lines with up to 32‐fold and 6‐fold greater efficiency than unmodified cholesterol‐containing LNPs at 6 h and 24 h post‐transfection, respectively [79]. ß‐Sitosterol is a C‐24 alkyl derivative of cholesterol, and other C‐24 alkyl derivatives were also shown to have similar effects on LNP transfection efficiencies in vitro. The observed improvement in LNP delivery is thought to be due to the structural and morphological effects of these cholesterol variants, since C‐24 alkyl derivatives result in the formation of highly faceted LNPs that may facilitate increased membrane fusion and endosomal bilayer disruption events relative to conventional LNPs, which tend to exhibit a more uniform curvature. Moreover, LNPs incorporating the modified cholesterol demonstrated higher rates of cell uptake and retention over 24 h, thus further enhancing levels of mRNA translation [79].

#### 2.2.4 PEG Lipids

PEGylated lipids provide LNP formulations with the benefits of colloidal stability, improved circulation time, and “stealth” properties, but they also offer a commensurate trade‐off in that they tend to reduce intracellular delivery efficiency [57]. This is because the PEG polymer chain grafted onto the end of the lipid creates a steric barrier that reduces the ability of fusogenic lipids on the LNP surface to interact with endosomal membranes [81]. In an attempt to reconcile these competing properties, most formulation strategies use PEG lipids at very low molar ratios (1%–2%) [57]. Furthermore, relatively short alkyl tails (e.g., 14‐carbon myristyl chains) are often used for the lipid portion of the PEGylating agent [57]. The lipid tails serve to anchor the PEG lipid into the LNP membrane, and thus a strong correlation exists between the length of the lipid tails and the strength of the membrane anchor [82]. Since PEG lipids are gradually shed from the surface of LNPs into circulation over time, shorter hydrophobic tails serve to accelerate the rate of shedding due to the weaker bonds that exist between the PEG lipid and the LNP relative to longer tailed PEG lipids [82]. Faster shedding enables improved cellular uptake and cytosolic delivery of payloads [83]. Third, the size of the PEG polymer chain can be reduced to enable greater interaction between fusogenic lipids and the endosomal membrane, thereby increasing LNP transduction efficiency [81].

The choice of PEG lipid also has important implications for tissue biodistribution patterns and immune responses. For example, Sago et al. observed that LNPs comprising 14‐carbon PEG lipids induced a differential tissue biodistribution pattern in mice compared to LNPs comprising 18‐carbon PEG lipids, with the former enriched in lung endothelial cells and the latter enriched in kidney endothelial cells [84]. The non‐trivial effects of PEG lipid properties on tissue biodistribution and delivery efficiency were also observed in separate work conducted by Dahlman et al. [85].

Concerningly, PEG lipids have been reported to induce rare, anaphylactic‐like immune responses in animal models and humans, with this potential side effect coming under increasing scrutiny due to the mass‐market approval of LNP‐encapsulated mRNA vaccines for the COVID‐19 pandemic [86, 87]. Beyond vaccines, potential PEG lipid‐induced immune responses are of even greater concern for monogenic diseases, where dosing is likely to be higher and administration more frequent. The mechanism by which this immune response is triggered is still to be fully elucidated [87], but is thought to be due to the production of anti‐PEG immunoglobulins (the exact isotype(s) considered pivotal to the immune response are still widely debated) [88, 89]. Interestingly, Suzuki et al. showed that LNPs comprising slow‐shedding PEG lipids (i.e., longer acyl chains) induced a much more pronounced IgM response in mice than LNPs formulated with fast‐shedding PEG lipids (i.e., shorter acyl chains), with the more immunogenic LNPs incurring greater sequestration by phagocytic Kupffer cells rather than target hepatocytes [83]. This finding suggests that precise tuning of the PEG lipid structure and molar ratio may be required in order to enable both safe and effective delivery of nucleic acids to target organs. Alternatives to PEG, such as polysorbate 80 [90] or polysarcosine [91], could also be explored.

### 2.3 Manufacturing and Quality Control

Particle‐formation technologies have progressed rapidly: high‐throughput microfluidic mixers now maintain tight residence‐time control and routinely generate 60–90 nm particles with polydispersity indices below 0.05, even when handling the high‐viscosity feeds required for circRNA or saRNA [92, 93]. At a larger scale, “flash‐nano‐complexation” and confined‐impinging‐jet systems preserve morphology in large‐volume batches, easing tech‐transfer to pandemic‐ready GMP suites. Once formed, key physicochemical attributes such as morphology, particle size, surface charge, and degree of surface shielding dictate biodistribution into different types of cells and tissues [94].

Formulation stability at elevated temperatures remains a bottleneck for multidose mRNA‐LNP regimens and depends on the exact formulation. Lyophilization provides long‐term stability for standard LNP formulations, but it is challenging for targeted LNPs that incorporate additional ligands on the LNP surface [95]. However, targeted LNP benefits from the multiple methods developed for surface functionalization: strain‐promoted azide‐alkyne cycloaddition enables post‐formulation attachment of transferrin‐receptor nanobodies or tandem‐dimer peptides without exposing ligands to shear or organic solvents, and these “clickable” LNPs markedly shorten process‐development timelines. Together, these architectural refinements have boosted tissue protein expression by one to two orders of magnitude since 2020, while aligning the platform with FDA and EMA expectations for biodegradability and chronic repeat dosing.

**FIGURE 2:**
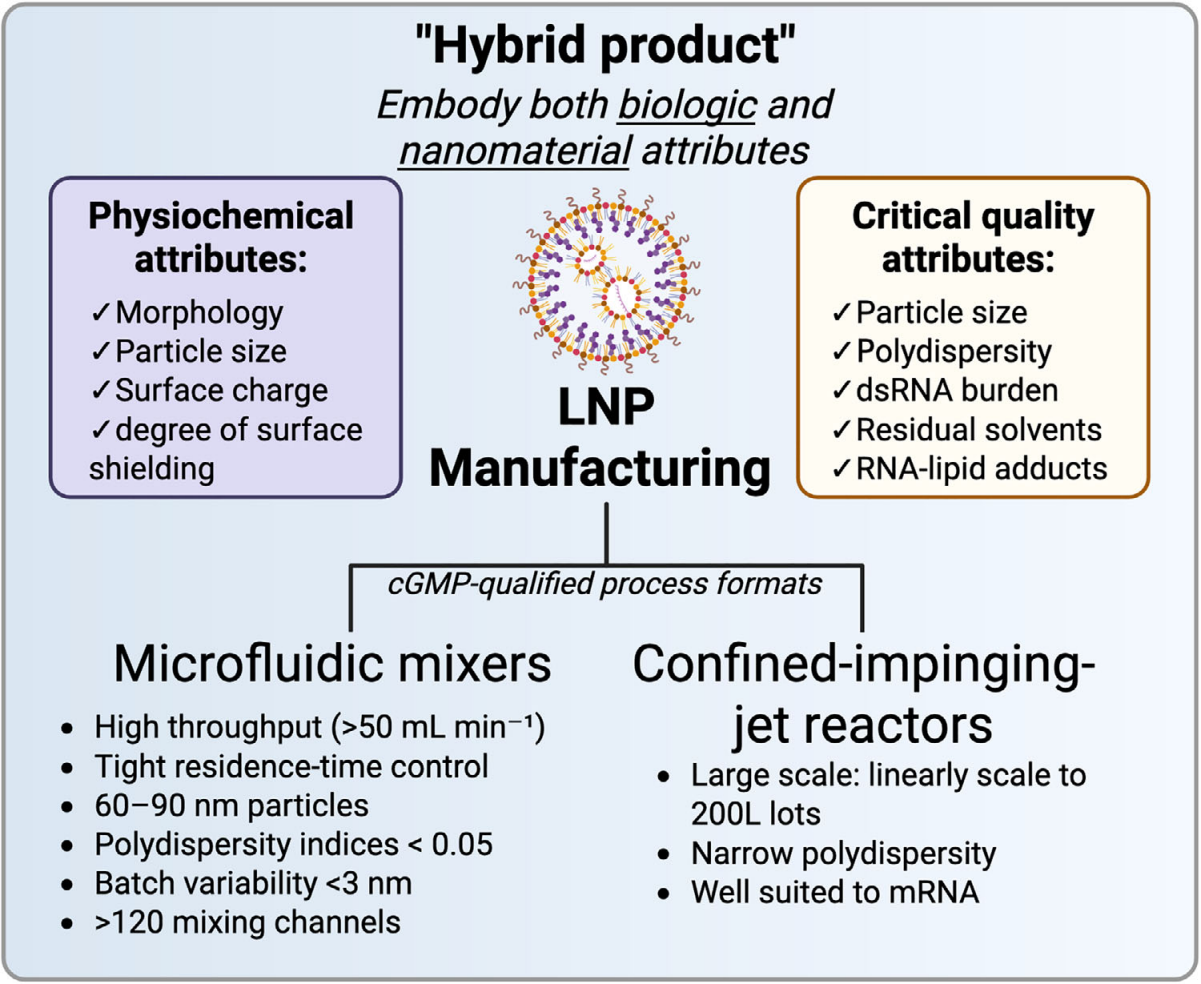
LNP manufacturing and classification. LNPs are classified by regulators as “hybrid products” and are characterized by a range of physicochemical attributes. Regulatory filings must demonstrate control over critical quality attributes, including encapsulation efficiency, particle size, polydispersity, double‐stranded RNA (dsRNA) burden, residual solvents, and RNA–lipid adducts. Manufacturing of LNPs is typically achieved through cGMP‐qualified processes, most commonly (1) microfluidic mixing or (2) confined‐impinging jet reactors, both designed to ensure consistent attainment of critical quality attributes.

Manufacture of LNP drug substance has diversified into two cGMP‐qualified process formats (Figure 2). Microfluidic mixers, prized for their tight residence‐time control, routinely deliver batch‐to‐batch variability of less than 3 nm in hydrodynamic diameter with existing technologies such as parallelized microfluidic devices capable of precise synthesis of LNPs using over 120 mixing channels that operate simultaneously [96]. Confined‐impinging‐jet reactors, by contrast, scale linearly to 200‐litre lots while preserving narrow polydispersity, and emerging “multicistern train‐in‐bag” lines, conceptually akin to single‐use bioreactors, provide low‐shear, high‐viscosity handling well suited to mRNA [97]. Regardless of the mixer, regulators regard mRNA‐LNPs as hybrid products that embody both biologic and nanomaterial attributes, so filings must satisfy a dual set of critical‐quality attributes covering encapsulation efficiency, particle size, polydispersity, dsRNA burden, residual solvents, and RNA‐lipid adducts. Real‐time release testing that marries inline dynamic light scattering with UV–Vis RNA quantification now compresses short quality‐control turnaround times, and increased stability data predict long shelf life for conventional formulations [98, 99]. Overlaying the physical plant is a digital quality infrastructure: AI‐enabled process‐analytical‐technology pipelines monitor hundreds of in‐process variables in real time, flagging excursions before they breach critical limits to maintain compliance with existing regulations.

## 3 The Ageing Blood–Brain Barrier and Neuroimmune Microenvironment

The physicochemical features of LNPs define how mRNA‐LNPs behave in circulation and within cells, but their performance in the CNS ultimately depends on how they interact with the ageing BBB. Age‐related changes in BBB structure and function, including altered tight‐junction integrity, reduced transporter availability, increased endothelial immune activation, and shifts in receptor expression, can modify nanoparticle trafficking and endosomal escape efficiency. Understanding these BBB‐specific constraints is therefore essential for translating general LNP design principles into effective formulations that can reach and transfect target cell types in the ageing brain.

**FIGURE 3:**
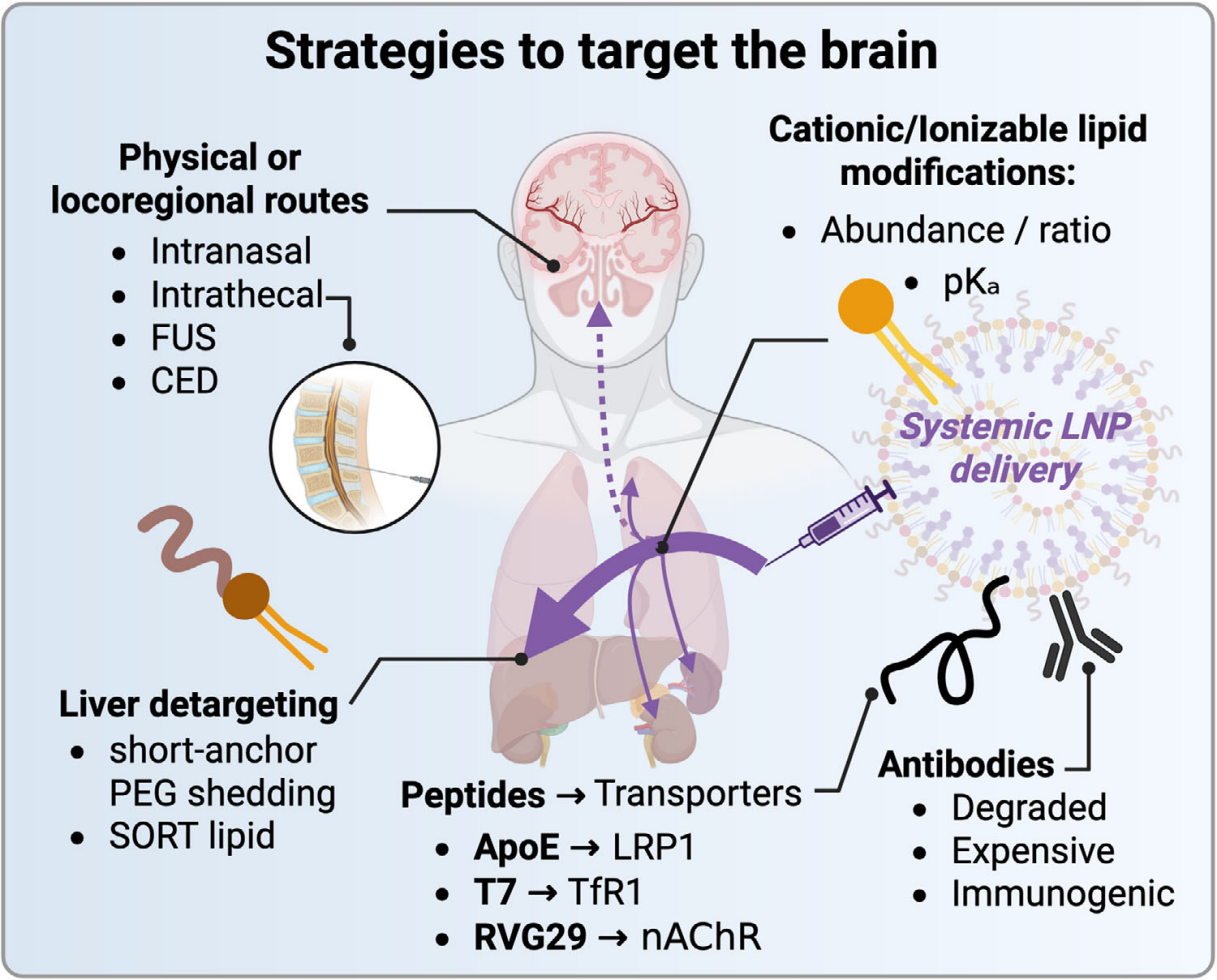
Strategies to target the brain and cross the blood–brain barrier (BBB). Approaches to enhance mRNA LNP delivery to the brain include modifying delivery techniques and routes, ranging from systemic administration to more direct physical or locoregional strategies (e.g., intranasal, intrathecal, focused ultrasound (FUS), or convection‐enhanced delivery (CED)). LNPs themselves can be engineered for tissue‐specific targeting by altering the abundance, ratio, and p*K*a of cationic/ionizable lipids. Because systemic LNP delivery is inherently biased towards the liver, de‐targeting strategies, such as short‐anchor PEG shedding and the use of selective‐organ‐targeting (SORT) lipids, have been developed to redirect nanoparticles to extrahepatic tissues. In addition, incorporation of short peptides or antibodies onto LNPs can further enhance BBB penetration and improve tissue specificity.

Ageing remodels the BBB through a combination of structural, molecular, and immunological changes that compound over decades [100, 101, 102] (Figure 3). Despite notable progress in drug‐development technologies, therapeutic success for brain disorders remains limited, largely because the BBB is still not fully understood and continues to impede the entry of most molecules, especially nucleic acids, which are hindered because of their large size and negative charge. Ageing exerts profound effects on BBB structure and function, and these changes are essential to consider when developing CNS‐targeted mRNA‐LNPs. In rodent studies, “aged” mice are typically defined as 18–24 months old (and 20–30 months in rats), age ranges that correspond to late‐life human stages with comparable vascular rarefaction, tight‐junction disruption, and heightened neuroinflammatory tone. Including such aged cohorts is therefore critical, as many BBB alterations relevant to neurodegeneration, including reduced transport capacity and increased immune priming, are not present in young adult animals.

The BBB's basic organization includes a layer of endothelial cells sealed by tight junctions and surrounded by pericytes and astrocytic end‐feet [103]. With advancing age and in neurodegenerative conditions such as AD and PD, this coordinated unit becomes progressively disrupted, leading to both structural and functional deterioration [104, 105, 106, 107]. Loss of tight junction proteins such as claudin‐5 and occludin, thickening and cross‐linking of the basement membrane, and progressive dropout of pericytes all contribute to impaired barrier integrity, increasing permeability and the likelihood of plasma protein leakage into brain parenchyma [102, 108]. These defects are closely linked to vascular damage that occurs in several neurodegenerative diseases [109]. Chronic hypoperfusion, oxidative stress, and microvascular rarefaction are hallmarks of the ageing cerebrovasculature and have been associated with both cognitive decline and BBB dysfunction [109]. Matrix metalloproteinases (MMP‐2 and MMP‐9) released by activated endothelial cells and glia further degrade tight junctions and basement membrane components, facilitating extravasation of fibrinogen, IgG, and other plasma proteins that exacerbate neuroinflammation [110]. Transporter expression is also altered: canonical receptors such as transferrin receptor 1 (TfR1), often exploited for nanoparticle delivery, show reduced expression or altered distribution in aged or diseased vessels [111], suggesting that nanoparticle formulations optimized in young animals may overestimate cortical delivery in the elderly.

Ageing also reshapes the neuroimmune milieu: Microglia adopt a chronically “primed” interferon‐responsive state, and border‐associated macrophages expand in number [112, 113, 114]. Both cell populations secrete cytokines such as IL‐6, TNFα, CXCL10, and CCL2, which amplify endothelial stress and may accelerate complement activation on PEG‐coated particles [112, 115]. Peripheral immune cells can also infiltrate more readily once tight junctions loosen, adding further heterogeneity to the inflammatory landscape [116, 117]. Collectively, these processes yield a barrier that is simultaneously leakier in some regions yet less permissive to receptor‐mediated trafficking and vesicular transport in others – a paradox that complicates the design of BBB‐penetrant nanomedicines.

Together, these spatial and cellular disparities mandate age‐stratified biodistribution studies and justify the routine inclusion of young and aged mice and other species when screening BBB‐crossing chemistries. Only by capturing the impact of vascular damage, transporter loss, and immune priming on delivery efficacy can nanoparticle formulations be credibly advanced towards clinical evaluation in the very populations where neurodegenerative disease burden is greatest [118]. For mRNA‐LNPs in particular, such age‐associated changes may shift the balance between effective delivery and immunotoxicity, underscoring the need for context‐specific design and evaluation.

## 4 Strategies to Cross the BBB With LNPs

### 4.1 Chemically Engineered Lipids

Ionizable lipids are the main structural component of every mRNA‐LNP. But more than just structure, their chemical nature enables them to condense polyanionic RNA in the acidic formulation buffer, present a near‐neutral surface at physiological pH to skirt opsonization, and then re‐protonate inside late endosomes to rupture the membrane and release the transcript [119, 120]. Although the core composition of mRNA‐LNPs is well established, small but systematic modifications to lipid chemistry, including ionizable headgroup structure, linker biodegradability, and PEG‐lipid tail length, can substantially influence particle pharmacokinetics and tissue distribution. These principles motivate high‐throughput lipid discovery approaches. For example, DNA‐barcoded LNP libraries, first developed for rapid mapping of tissue‐selective nanoparticles in vivo, represent a potential strategy for future identification of lipid chemistries that favour BBB engagement and brain transfection, even though such barcoding has not yet been applied specifically to CNS‐directed mRNA‐LNPs [85].

A complementary discovery route has been demonstrated by Wang et al., who performed multi‐stage in vivo screening and structure–activity optimization to identify a new class of ionizable lipids capable of crossing the BBB after systemic administration. Their optimized MK‐series lipids supported delivery of mRNA to both neurons and astrocytes across widespread brain regions. In particular, biodistribution analysis of fluorescently labelled MK16 BLNPs showed that ∼15% of the total LNP fluorescent signal was detected in the brain after intravenous administration, while functional reporter studies demonstrated ∼6‐ to 7‐fold higher protein expression in the brain when compared to benchmark LNP formulations [121]. This work highlights how rational design and iterative screening can yield chemotypes with emergent CNS‐targeting properties [121].

Because only a very small fraction of systemically administered LNPs cross the BBB, efforts to design brain‐directed formulations must focus on improving the efficiency of the particles that do enter the CNS while minimizing peripheral toxicity. Rather than attributing BBB permeation to any single structural motif, recent work suggests that multiple parameters, including ionizable lipid p*K*_a_, linker degradability, PEG–lipid architecture, and tail branching, collectively shape biodistribution and tolerability [67, 122, 123, 124, 125]. These features influence how long LNPs circulate, how they interact with serum proteins, and how they escape the endosome once inside CNS cells. Importantly, increases in endosomal‐escape efficiency or reductions in hepatic uptake can both raise the amount of functional protein expressed in the brain without requiring proportionally higher systemic doses. As such, optimizing ionizable lipid chemistry is less about identifying a universal “BBB‐shuttling” motif and more about balancing expression in the CNS with safety across peripheral organs. This framing also aligns with subsequent screening studies such as the MK‐series work by Wang et al., which identified BBB‐active chemistries by iterative optimization rather than by relying on any single structural rule [121].

Three current challenges confront the clinical translation of today's BBB‐shuttling lipids. First, delivery efficiencies observed in rodents do not necessarily translate to larger brains, where barrier properties and transport mechanisms differ across species [102]. Second, the synthetic complexity of many ionizable lipids constrains library size and scalability, though advances in flow chemistry and AI‐guided retrosynthesis are beginning to ease this bottleneck [122]. Third, the ionizable lipid itself often defines the safety ceiling: cationic head groups have been linked to hepatotoxicity and complement activation, and maximum tolerated doses in clinical programmes are generally determined by lipid exposure rather than by the RNA cargo [126]. Efforts to introduce biodegradable linkers can mitigate these toxicities, but very rapid clearance may also reduce formulation stability, forcing a balance between tolerability and shelf life [126].

### 4.2 Ligands and Peptide‐Functionalized LNPs

One of the most widely used methods to compensate for the tissue biases of ionizable lipid chemistry in mRNA‐LNPs is to cloak the particle in ligands that hijack receptor‐mediated transcytosis or adsorptive endocytosis, a “bolt‐on” strategy that works with virtually any mRNA cargo. In practice, ligands are attached after particle formation, typically by maleimide‐thiol coupling or strain‐promoted azide‐alkyne “click” chemistry, to avoid exposing delicate receptor‐binding epitopes to organic solvent [127]. A 2–3 kDa PEG spacer helps keep the binding moiety accessible while preserving colloidal stability, and ligand density must be tuned, because crowding the surface can saturate receptors and shunt particles into non‐productive uptake pathways. A further, often‐overlooked hurdle is orientation: conventional maleimide‐thiol chemistry randomly grafts cysteines around the ligand's surface, so only a fraction of the copies stand “upright” and available for binding. Site‐specific approaches (e.g., sortase‐tagging, engineered single cysteines, or orthogonal click handles) can impose the desired orientation but require additional protein engineering and process steps, adding another layer of complexity to scaled manufacture [128].

One such functionalization strategy involves the attachment of antibodies on the surface of LNPs, which allows targeting of specific tissues such as lungs [129, 130] or specific cell types such as cancer cells [131, 132] (Figure 3). Although antibodies offer excellent binding affinity, they come with notable drawbacks: they are prone to proteolytic degradation, can provoke immune responses, require precise orientation to bind effectively, and are costly to manufacture because of their large, complex structure [133, 134]. Short peptides (typically 30 amino acids or fewer) circumvent many of these limitations [135]. Their small size allows a higher ligand density on the nanoparticle surface, which can enhance avidity, and they are often less immunogenic, more protease‐resistant when suitably modified, and far cheaper to produce than full‐length antibodies [136].

Most peptide‐based functionalization studies that focused on neurological applications target receptors that are abundantly expressed at the BBB, including TfR1 and apolipoprotein E (apoE) receptors such as LRP1 [137, 138] (Figure 3). A recent study by Han et al. involved designing a peptide‐functionalized LNP platform for targeted mRNA delivery to the brain and characterizing several LNPs functionalized with different peptides corresponding to RVG29, T7, AP2, and apoE [139]. The study identified a lead RVG29 LNP formulation with a 10% lipid‐PEG‐maleimide that achieved high neuronal expression with low entrapment in BBB endothelial cells [139].

Remaining challenges cluster around cross‐species affinity, endosomal escape, manufacturability, and cell‐type specificity within the CNS. Most BBB‐penetrant LNPs that achieve parenchymal delivery are preferentially taken up by glial cells, leaving neuronal transfection comparatively sparse [140]. Developing strategies that either bias uptake towards neurons or balance expression across multiple CNS cell types will be critical for broadening the therapeutic scope of these formulations. Many ligands designed to engage receptors at the BBB exhibit variable efficacy across species [111]. Because uptake alone does not ensure cytosolic release, dual decoration with fusogenic motifs such as the glutamic‐acid–alanine–leucine–alanine peptide or the influenza haemagglutinin–derived INF7 is under active investigation [141, 142]. Attaching ligands after particle formation introduces an extra unit operation along with additional controls and analytics [143]. Even so, peptide‐ or antibody‐decorated LNPs remain the most versatile receptor‐guided strategy for crossing the BBB, and they have delivered the strongest biodistribution profiles seen so far in large‐animal studies [139, 140]. The outstanding tasks for ligand‐decorated LNP platforms are to demonstrate consistent performance across species and to integrate ligand coupling into a streamlined, economically viable manufacturing process.

### 4.3 Liver‐Detargeted Formulations

Although liver‐detargeting is not a BBB‐crossing mechanism, it remains relevant for CNS‐focused formulations because the liver sequesters most systemically administered LNPs [144]. Reducing hepatic uptake can increase the circulating fraction available for extrahepatic delivery and lower dose‐limiting liver toxicity, a key consideration when higher systemic doses may be required for therapeutic levels to reach the brain. Several chemistry‐based strategies have been explored to modulate liver capture. Adjusting PEG–lipid kinetics, for example by varying anchor length or increasing PEG density, suppresses apoE binding and slows hepatic clearance [144, 145, 146]. Polysarcosine‐functionalized lipids reproduce PEG's hydrophilicity while reducing complement activation and anti‐PEG antibody formation [147]. A complementary approach, the “selective‐organ‐targeting” (SORT) paradigm, alters the lipid core composition by incorporating a fifth charged lipid to redistribute expression towards the lung or spleen [73]. However, while these methods effectively redirect LNPs away from the liver, they do not on their own facilitate BBB transport, and additional ligand‐based or chemistry‐based strategies are required for brain targeting [148]. Together, these findings indicate that liver‐detargeting modules, whether via PEG tuning, PEG alternatives, or SORT chemistry, may serve as enabling components for future CNS‐directed LNPs, improving tolerability and increasing the fraction of dose available for brain delivery [149].

### 4.4 Physical or Locoregional Routes

Although most programmes focus on systemic administration, Tuma et al. demonstrated that a clinically approved DLin‐MC3‐DMA formulation can sustain eGFP translation for 3 weeks after a single stereotaxic injection, indicating that direct parenchymal dosing remains a viable option for focal diseases or device‐assisted delivery [150]. While systemic administration remains the ultimate goal for most neurodegenerative indications, a growing toolkit of locoregional and physically assisted delivery techniques can either supplement or temporarily replace intravenous dosing while BBB‐penetrant chemistries mature.

**Focused ultrasound (FUS)** coupled with microbubbles has progressed in recent years. FUS‐BBB opening couples a low‐duty 3 MHz acoustic beam with intravenously injected microbubbles; the oscillating bubbles generate transient, millimetre‐scale pores in the endothelial tight junctions, allowing LNPs to cross the BBB [151]. In the Ogawa et al. mouse study, a single 60 s sonication at 1.5 kW cm^−^^2^ targeted to the right striatum immediately after tail‐vein administration of both luciferase‐mRNA LNPs and microbubbles opened the barrier without detectable haemorrhage or oedema [152]. This brief window was sufficient to boost luciferase protein expression on the irradiated side, while the contralateral hemisphere remained unchanged, confirming spatial precision. Immunofluorescence experiments revealed that the translated protein localized predominantly to CD31^+^ endothelial cells and Iba1^+^ microglia, with no signal in astrocytes and neurons, indicating that FUS‐enabled entry favours vascular and immune interfaces rather than parenchymal neurons under these conditions [152]. Together, these data show that FUS + microbubbles can convert peripherally dosed mRNA‐LNPs into region‐restricted, cell‐selective protein expression in the brain, without producing tissue damage. In disorders such as AD, however, where the BBB is already compromised [100, 153, 154], the incremental benefit of FUS may narrow, and safety margins will need to be re‐established in tissue that is both leakier and more vulnerable to mechanical stress.

**Intrathecal (lumbar) administration** delivers LNPs directly into cerebrospinal fluid, bypassing endothelial barriers altogether and placing the particles in immediate contact with dorsal‐root‐ganglion (DRG) somata and the caudal spinal cord (Figure 3). In the study by Han et al., a nine‐member panel of DLin‐MC3‐DMA LNPs was injected in 5 µL volumes; the formulation with the smallest hydrodynamic diameter (≈55 nm) and a negatively charged DMG‐PEG‐COOH corona achieved markedly higher accumulation of Cy5‐siRNA in lumbar DRGs compared with free oligonucleotide and outperformed larger or cationic counterparts [124]. This same “55 nm” formulation was also transported retrogradely along axons and produced a rapid, reversible reduction in TRPV1 mRNA in vivo with substantial knockdown evident at 48 h and full recovery by four weeks [124]. Collectively, these data show that lumbar intrathecal delivery can achieve selective, reversible gene silencing in sensory neurons without detectable systemic or local toxicity, underscoring its promise as a complementary route for CNS‐adjacent mRNA or siRNA therapeutics.

**Intranasal instillation** takes advantage of the direct anatomical channels linking the nasal mucosa to the olfactory bulb and trigeminal nerves, bypassing first‐pass metabolism and the endothelial tight junctions of the BBB. Formulation studies show that polymeric or lipid nanocarriers <200 nm, carrying a mildly positive charge and coated with muco‐penetrating or muco‐adhesive polymers, can linger on the nasal epithelium, resist enzymatic degradation, and traverse the epithelium more efficiently [155, 156]. Yet important caveats temper rodent‐based successes. The murine nasal cavity is proportionally much larger (1/3 of the brain volume), more vascularized, and more richly innervated than its human counterpart, so dosing volumes, deposition patterns, and clearance kinetics do not scale linearly [157, 158]. Many mouse and rat protocols rely on deep anaesthesia and pipette instillation [159]: conditions that hold droplets against the olfactory roof far longer than would occur in an ambulatory person with active mucociliary clearance. In humans, the usable instillation volume is limited, a substantial fraction is swallowed, and variability in sniff strength, head position, and nasal pathology further erodes dose precision [160]. These translational gaps must be addressed through device design, formulation tuning, and human‐relevant airflow models before intranasal LNPs can be considered a reliable stand‐alone route for CNS mRNA therapeutics. Clinical validation of the pathway already exists: intranasal esketamine (Spravato) was approved in 2019 for treatment‐resistant depression, demonstrating that therapeutically relevant concentrations can be achieved in the brain via this non‐invasive route, although mRNA‐LNP delivery is likely more challenging due to size differences compared with the small molecule drug [161, 162, 163].

Pre‐clinical data with lipid‐based systems reinforce the translational potential of intranasal delivery for CNS therapeutics. Solid‐LNPs or PLGA particles given intranasally have repeatedly raised brain drug levels above those obtained by intravenous administration and have shown functional benefit in disease models [164]. For example, lamotrigine encapsulated in PLGA nanoparticles reached higher cerebral concentrations and produced superior seizure protection after intranasal dosing compared with both IV nanoparticles and aqueous formulations in rats; similarly, agomelatine‐loaded solid‐LNPs delivered through the nose achieved greater brain exposure than commercial oral tablets and IV drugs in rodents [165]. Collectively, these findings indicate that, once formulation parameters such as particle hydrophobicity, muco‐penetration, and release kinetics are optimized, intranasal LNPs could present as a minimally invasive adjunct or alternative to systemic or intrathecal dosing for mRNA therapeutics targeting neurodegeneration.

**Convection‐enhanced delivery (CED)** offers focal, high‐concentration dosing via a stereotactically placed cannula. Pressure‐driven perfusion generates a bulk flow “cloud” that can cover entire basal ganglia nuclei, attractive for PD gene therapy [166]. The study by Waggoner et al. (2023) applied CED to deposit 3.5 µL of Cy5‐labelled mRNA‐LNPs into the mouse striatum. Serial coronal sections revealed that the particles spread over 1 mm from the cannula tract, demonstrating that convective forces can move LNPs far beyond the limits of simple diffusion. Confocal microscopy showed robust colocalization of the fluorescent signal with NeuN‐positive neurons, Iba1‐positive microglia, and GFAP‐positive astrocytes, confirming that CED exposes all major parenchymal cell types to the mRNA cargo. Together, these findings establish CED as a practical intra‐parenchymal route for distributing mRNA‐LNPs within a reasonable vicinity of the injection site while engaging diverse brain‐resident cells [167].

Collectively, these physical and locoregional strategies provide valuable bridging technologies: they can validate therapeutic hypotheses in large brains, deliver high local payloads for focal diseases such as glioma, and, in the case of FUS, may ultimately combine with BBB shuttling chemistries for fully non‐invasive, repeated dosing. A concise comparison of the opportunities, challenges, and limitations of each BBB‐targeting strategy is provided in Table 2, which highlights how chemically engineered lipids, ligand‐directed formulations, and physical or locoregional methods differ in mechanism, efficiency, and translational constraints.

**TABLE 2:** Comparison of BBB‐targeting strategies for mRNA‐LNP delivery.

Strategy	Mechanism of BBB interaction	Representative examples	Reported delivery, transfection outcomes	Primary brain cell types targeted	Major limitations
**Chemically engineered ionizable lipids**	BBB transit enabled by optimized lipid chemistry (p*K*_a_, tail branching, linker degradability), improved circulation, and increased CNS parenchymal uptake	MK‐series ionizable lipids (e.g., MK16)	Systemic administration results in detectable mRNA expression in neurons and astrocytes across multiple brain regions	Neurons, astrocytes	Not validated in large animals; low fraction of injected dose reaches brain
**Ligand‐ or peptide‐functionalized LNPs**	Receptor‐mediated transcytosis across BBB endothelial cells via ligands or peptides targeting TfR1, LRP1, insulin receptor, or apoE receptor motifs	Transferrin receptor–binding peptides; RVG29 peptide; apoE‐mimetic peptides; antibody‐functionalized nanocarriers	Enhanced endothelial uptake and improved CNS distribution in rodents; expression dependent on ligand affinity and receptor expression	Endothelial cells, neurons and glia (secondary to transcytosis)	Receptor saturation; species differences in receptor expression; immunogenicity of antibodies/peptides
**Physical and locoregional methods**	Mechanical or spatial BBB modulation to permit entry of LNPs into targeted CNS regions	Focused ultrasound (FUS) + microbubbles; intrathecal/intracisternal delivery; convection‐enhanced delivery	FUS enables focal entry of LNPs into parenchyma; intrathecal routes result in distribution along the neuraxis; efficiency depends on dosing route and method	Region‐specific neuronal and glial uptake (FUS); broad CNS coverage (intrathecal)	Requires specialized equipment or invasive delivery; difficult to scale for repeated dosing; safety considerations for FUS
**Liver‐detargeted formulations (enabling strategy)**	Reduced hepatic uptake increases circulating fraction available for CNS delivery and improves tolerability for systemic dosing	PEG‐density tuning; PEG–anchor length variation; polysarcosine‐coated LNPs; SORT lipids	Redistribution towards extrahepatic organs; not a BBB‐crossing mechanism by itself	Not CNS‐specific; increases systemic exposure	Must be combined with BBB‐targeting chemistries; does not induce BBB transcytosis

### 4.5 Comparative Analytics

Rigorous, multimodal analytics are now essential for judging BBB‐crossing strategies because they must reveal not just how much protein is made, but where, when, and in which cell type the mRNA is translated. Early lipid screens leaned heavily on whole‐brain luciferase bioluminescence imaging, a fast and sensitive metric that integrates total enzyme activity yet provides no spatial context and can wildly overestimate neuronal delivery when endothelial and meningeal expression dominate the photon flux. To tease these compartments apart, many groups have switched to Cre‐loxP reporter mice: an mRNA‐encoded Cre recombinase excises a stop cassette upstream of tdTomato or GFP, and subsequent histology counts fluorescent neurons, astrocytes, and microglia one by one [168]. Reporter genetics are now complemented by single‐nucleus RNA‐seq and spatial transcriptomics, which map mRNA‐derived reads to precise coordinates and deconvolute lipid libraries across different brain cell types. High‐throughput in vitro screening platforms are also catching up: Han et al. developed a high‐throughput in vitro screening platform that simultaneously assesses mRNA LNP transfection and transport across the BBB and demonstrated that the method can predict in vivo delivery of mRNA‐LNP to the brain [169].

Non‐terminal imaging now helps bridge temporal gaps in screening for mRNA uptake. MRI chemical‐exchange saturation‐transfer can detect the exchangeable imino protons of pseudouridine‐rich mRNA, enabling in situ visualization of transcript distribution in living animals; the method remains pre‐clinical but has resolved on‐target expression in mouse cortex within hours of dosing. Fluorine‐18 (^18^F)‐labelled helper lipids provide a complementary positron‐emission‐tomography handle for the particle itself [170]. During lead optimization, the imaging data are analyzed alongside standard immunogenicity panels (serum cytokines, complement split products and anti‐PEG antibody titers), and early machine learning models that ingest all these features may be used to flag efficacy‐toxicity “sweet spots”. Taken together, this layered toolkit of cellular reporters, multi‐omics, live imaging, and immune readouts has become essential for distinguishing formulations that merely reach the brain from those that deliver therapeutically meaningful expression in the right cell populations.

## 5 Preclinical Efficacy Landscape

Since 2021, brain‐directed mRNA‐LNP work has progressed from isolated demonstrations to a modest corpus of ≈30 peer‐reviewed studies reporting functional protein expression in rodent brain parenchyma [171]. Although the field still lacks a single harmonized efficacy metric, most laboratories now triangulate performance with (i) absolute protein or enzyme activity in brain lysate and/or target brain cell populations, (ii) biochemical, behavioural, or electrophysiological rescue in disease models, and (iii) post‐mortem counts of reporter‐positive cells [171].

The most detailed dataset comes from the Wang et al. study that identified MK16 as a lead chemotype in mice, achieving neuronal and astrocytic transfection after a single intravenous mRNA‐LNP dose, with expression levels 6‐ to 7‐fold higher than benchmark LNPs [121]. ApoE‐modified LNPs have also been tested systemically in mice, yielding endothelial uptake, although still representing a small fraction of the injected dose (<1% following intravenous administration), highlighting persistent endosomal escape and trafficking barriers [172]. Complementing these ligand‐based approaches, FUS combined with LNPs achieved neuronal GFP expression in the mouse hippocampus after systemic dosing, suggesting that acoustic co‐modalities may further enhance delivery efficiency in rodent models [173]. Although several studies report measurable neuronal and astrocytic transfection following systemic administration of optimized ionizable lipid formulations, the therapeutic meaning of any given percentage of transfected cells or protein‐expression level is highly context‐dependent. The degree of expression required for benefit can vary markedly between disease categories; for example, partial restoration of enzyme activity may be sufficient in metabolic or loss‐of‐function disorders, whereas neuroprotective factors or modulatory receptors may exert biological effects even when expressed in only a small subset of cells.

To date, published demonstrations of brain‐directed mRNA‐LNP expression remain largely confined to rodent models, and no peer‐reviewed study has yet shown parenchymal mRNA translation in non‐human primates following systemic LNP delivery. Nevertheless, related work in large animals provides important translational insights. First, ionizable lipid formulations similar to those used for CNS‐targeted LNPs in rodents have demonstrated sustained protein expression and favourable safety in non‐human primates after intravenous or intramuscular delivery, which demonstrates that the underlying mRNA‐LNP chassis scales safely to larger species [24, 174]. Second, intrathecal administration of LNPs has been shown to achieve efficient delivery across the neural axis, with 100‐ to 200‐fold enrichment of siRNA in dorsal root ganglia relative to free siRNA and predominant accumulation in the spinal cord and dorsal root ganglia, peaking at 6–12 h post‐administration, thereby providing a quantitative benchmark for CNS‐targeted LNP formulations [124]. Third, ligand‐based BBB targeting strategies validated in rodents, such as transferrin receptor and apoE receptor pathways, are known to remain functional in primates, as established by receptor biology studies and nanoparticle transport experiments using non‐mRNA cargos [111, 138]. Together, these findings highlight that while rodent models remain essential for rapid screening, robust evaluation in aged and large‐brain species will be required to establish safety, dose–exposure relationships, and expression durability before advancing CNS‐targeted mRNA‐LNPs towards first‐in‐human studies.

Because most neurodegenerative phenotypes and BBB impairments emerge only in aged animals, age‐stratified biodistribution and efficacy studies (young vs. aged cohorts) should become standard practice for CNS‐directed mRNA‐LNP development. This is especially important given that the intended patient populations for AD, PD, and related disorders are predominantly elderly, and rodent efficacy results obtained only in young animals may overestimate translational potential. A structured overview of age‐related alterations in the BBB and their implications for mRNA‐LNP delivery is provided in Table 3, which summarizes how vascular, immune, and transport changes in older animals influence biodistribution, endosomal trafficking, and the likelihood of achieving functional expression in the ageing brain.

**TABLE 3:** Age‐related changes in the BBB and their implications for mRNA‐LNP delivery.

BBB feature	Age‐related change	Impact on mRNA‐LNP delivery	Implications for therapeutic design
Tight junction between endothelial cells	Decreased expression of tight‐junction proteins such as claudin‐5, occludin, and ZO‐1; structural disorganization in older animals and humans	Increased paracellular leakiness but inconsistent across regions	Screening should include multiple brain regions and aged models; uniform permeability in the elderly brain should not be assumed
Transcytosis across endothelial cells	Increased caveola‐dependent transport with age; altered vesicle trafficking in older tissue	Greater nonspecific uptake into endothelial cells, low targeting of neurons and glia	Reinforces the need for formulations that minimize escape in endothelium but promote escape in brain cells (“switchable escape”)
Transporter expression at the BBB	Reduced levels of glucose transporter GLUT1, lipoprotein receptor–related protein 1 (LRP1), and other key transporters in old age	Decreased efficiency of ligand‐mediated targeting approaches relying on these receptors	Ligand‐coated LNPs must be tested in aged models; successful targeting in young mice may not translate
Innate immune activation in the endothelium	Elevated baseline production of interferons and inflammatory cytokines; endothelial cells exhibit “primed” immune states	Greater sensitivity to LNP components, increasing risk of inflammatory responses at the barrier	Encourages development of less immunogenic lipid chemistries, optimized dosing schedules, and careful evaluation of immune activation in older models
Pericyte coverage and function	Reduced pericyte number and altered morphology; impaired ability to regulate barrier stability	Weaker barrier integrity and reduced ability to respond to cellular stress	Greater variability in LNP penetration across individuals; emphasizes screening in physiologically relevant, age‐appropriate systems
Changes in the extracellular matrix and basement membrane	Thickening, stiffening, and altered composition of extracellular matrix proteins	Reduced diffusion of nanoparticles and altered interactions with vascular basement membranes	Nanoparticle designs may require optimized size, surface chemistry, or design to move efficiently through aged extracellular environments

Safety findings across the published animal studies remain broadly optimistic but closely linked to the nature of the ionizable lipid. In most studies, the acute reactogenicity profile mirrors that seen with authorized mRNA vaccines: transient pyrexia, modest interleukin‐6 rises that normalize within 24–48 h, and a brief neutrophilia [6, 175]. Serum ALT and AST rarely stray outside the reference range even after cumulative systemic doses, and complement‐activation‐related pseudo‐allergy has been reported only in swine studies that used very high infusion rates [176, 177]. Expression durability is mRNA‐ and cell type‐dependent, so maintenance schedules will need to reflect that divergence [178, 179]. Anti‐drug antibodies, including anti‐PEG IgG, remain negligible through the repeat doses evaluated so far [24, 174]. Overall, the published evidence paints a platform that is potent and safe in rodents, partially effective in mid‐size brains, and still searching for the formulation‐delivery method pairing that will unlock robust, cell‐specific delivery in human‐scale cortex without sacrificing the promising safety profile observed for newer generations of IV formulation mRNA therapeutics.

## 6 Lessons From Systemic mRNA‐LNP Therapies

mRNA‐LNPs have already been administered to well over a billion individuals as COVID‐19 vaccines [6] and, more recently, to several dozen patients in first‐in‐human studies for rare metabolic disorders (clinical trials: NCT04899310; NCT04159103) [174]. Although none of these trials target the CNS, their cumulative datasets provide a blueprint for brain‐directed programmes.

### 6.1 Clinical Proof‐of‐Concept Beyond Vaccines

The first generation of non‐vaccine mRNA therapeutics for indications other than oncology focuses on inborn errors of metabolism where secreted or intracellular enzymes are missing. Moderna's mRNA‐3927 was developed to deliver propionyl‐CoA carboxylase subunits to hepatocytes as a treatment for propionic acidaemia. In an open‐label, dose‐escalation trial of mRNA‐3927 for propionic acidaemia, patients receiving bi‐weekly 0.5 mg kg^−^^1^ infusions showed marked reductions in toxic metabolites (2‐methylcitrate and 3‐hydroxypropionate), which were accompanied by fewer episodes of metabolic decompensation [174]. Meanwhile, mRNA 3705 for methylmalonic acidaemia has cleared the US FDA IND and is dosing adults in an open‐label, ascending‐dose study (NCT04899310), and mRNA 3351 for glycogen storage disease 1a has cleared the IND and is dosing adults [170].

### 6.2 Safety and Immunogenicity Landscape

Across these studies, the predominant adverse events are grade 1–2 flu‐like symptoms (pyrexia, myalgia) resolving within 48 h. Grade ≥3 laboratory abnormalities are rare (<5 %) and include transient ALT/AST elevations. Myocarditis/pericarditis, a concern after high‐dose Spike vaccines, remains confined to adolescent males and appears linked to dosing interval rather than cumulative lipid exposure [180]. Comprehensive immune profiling in vaccinated adolescents and young adults showed no discernible differences in the overall mRNA‐vaccine response between those who later experienced myocarditis and those who did not [181]. What did set the myocarditis group apart was the presence of circulating, unbound spike protein in their plasma, a finding that offers a new clue into the possible mechanism underlying post‐mRNA‐vaccine myocarditis [181].

### 6.3 Manufacturing, Logistics and Cost Lessons

The COVID‐19 scale‐up proved that end‐to‐end timelines of <12 months from sequence to licensed product are achievable when regulatory pathways are well mapped and supply chains are secured [4, 182]. Throughput is still constrained by a limited but steadily expanding pool of GMP‐grade ionizable lipid suppliers; post‐pandemic investment by multiple contract manufacturers has begun to ease the single‐source bottleneck, yet long‐term capacity and redundancy remain works in progress. Dual sourcing of lipids, distributed modular GMP suites and initiatives such as the WHO mRNA Technology Transfer Programme in South Africa and other countries are helping to mitigate this bottleneck (source: WHO mRNA Technology Transfer Program; mRNA TT). For CNS programmes, smaller batch sizes suffice, but sterility and RNA integrity standards are identical; thus, platform processes can be reused with minimal tech transfer friction. Independent techno‐economic analyses estimate that, at a commercial scale, drug‐substance and fill‐finish costs for mRNA‐LNP products lie in the low single‐digit US‐dollar range per dose, with capping reagents, high‐purity nucleotides, and chromatographic resins accounting for the bulk of the expense [183, 184].

### 6.4 Regulatory and Reimbursement Precedents

The EMA and FDA now recognize “platform experience” to justify lighter non‐clinical packages for new payloads that use an established LNP chassis. Favourable toxicology reports from COVID‐19 vaccines and Moderna's rare disease mRNA‐LNP repetitive dosing trials could accelerate patient dosing and recruitment for CNS diseases. Value‐based pricing arrangements are increasingly being explored for rare diseases, particularly with gene therapies, and these frameworks are expected to set reimbursement precedents as mRNA therapeutics such as those for inborn errors of metabolism move towards the clinic [185, 186]. Such models may be particularly relevant to CNS diseases, where biomarker‐driven endpoints may substitute for long‐term cognitive outcomes.

### 6.5 Transferable Insights for CNS Programmes

**FIGURE 4:**
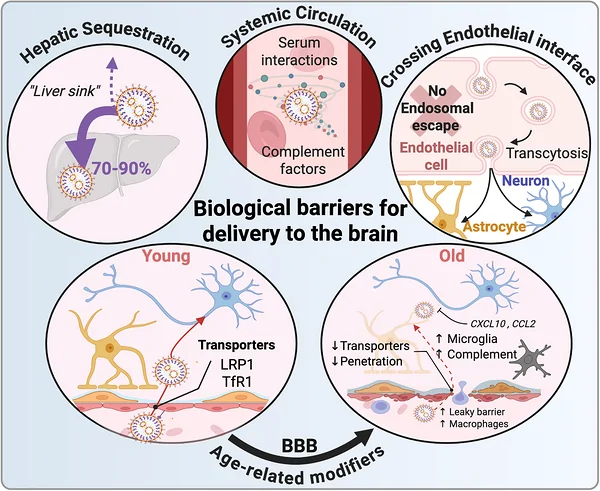
Biological barriers for systemic mRNA‐LNP delivery to the brain. Brain‐targeted mRNA‐LNPs face multiple biological filters: serum interactions, hepatic uptake, and endothelial barriers before reaching brain cells. The BBB also undergoes structural and functional remodelling with ageing and disease. Key changes include reduced barrier integrity with increased permeability and leakage, decreased expression of transporters (e.g., transferrin receptor, TfR1; low‐density lipoprotein receptor‐related protein 1, LRP1), and an altered immune environment characterized by interferon‐responsive microglia, expansion of border‐associated macrophages, and secretion of cytokines (e.g., CXCL10, CCL2) that can accelerate complement activation. mRNA‐LNP designs must therefore balance biodistribution, immunogenicity, and compartment‐specific endosomal escape efficiency for efficient delivery of therapeutic mRNA to target cells in the brain.

Clinical experience with systemic mRNA therapeutics offers several transferable lessons for brain‐directed programmes. First, repeated dosing is clearly feasible: the two‐to‐three‐week infusion cadence used in metabolic‐liver trials has been well tolerated and fits the long‐term treatment regimen expected for progressive neurodegenerative disorders [174]. For context, children with CLN2 Batten disease currently receive cerliponase alfa every other week by direct intracerebroventricular infusion – an invasive, operating‐room procedure [187]. The fact that mRNA‐LNPs can be given with similar frequency intravenously underscores their potential to deliver CNS‐active proteins or enzymes without the surgical burden that today's enzyme‐replacement therapies impose [187]. Second, the manufacturing infrastructure that sprang up during the pandemic can ramp production up quickly when needed, but it can also slow down or shut off just as easily when demand drops. A visual overview of the biological barriers confronted by systemically administered mRNA‐LNPs is provided in Figure 4, which highlights that CNS‐targeted formulations face all the same pharmacokinetic and innate‐immune constraints as systemic mRNA‐LNPs but encounter additional challenges, including the extremely low fraction of particles that penetrate the BBB, the disproportionately higher doses required to reach brain parenchyma, and the resulting trade‐off between CNS exposure and peripheral toxicities such as liver inflammation.

Equally important are the safety and societal dimensions. Digital pharmacovigilance networks that first flagged rare myocarditis cases after COVID‐19 vaccination can be repurposed to monitor neuroinflammatory signals during early CNS trials, providing regulators with near‐real‐time data and sponsors with an early‐warning system. Finally, the unprecedented scale of the COVID‐19 rollout has made mRNA a familiar term to regulators and the public, which should, in principle, ease ethics committee reviews and speed recruitment for dementia trials. At the same time, the pandemic also exposed a persistent strain of vaccine hesitancy often amplified by misinformation that conflates all “mRNA products” with vaccination. Sponsors will therefore need clear, proactive communication that distinguishes transient, non‐integrating mRNA therapeutics from immunization, emphasizes rigorously monitored safety data, and engages patient‐advocacy groups early. Addressing that perception gap up‐front is critical if the platform's regulatory momentum is to translate into broad patient acceptance.

## 7 Translational Roadmap and Regulatory Considerations

Bringing a BBB‐penetrant mRNA‐LNP from academic proof‐of‐concept to first‐in‐human dosing has the potential to become a more scalable and faster development process than for small molecules because both the lipid chassis and the in vitro‐transcribed RNA process are now recognized as modular “platform” technologies, but it is also more intricate, owing to dual oversight that treats the product simultaneously as a biologic and as a nanomaterial. Success demands that regulatory strategy, pre‐clinical evidence, CMC maturity, and stakeholder alignment advance in lockstep rather than sequentially.

### 7.1 Global Regulatory Landscape

Moving a BBB‐penetrant mRNA‐LNP from bench to first‐in‐human testing should become faster than the traditional small‐molecule route because both the lipid chassis and the in vitro RNA process are increasingly viewed as platform technologies. The journey remains complex: regulators often evaluate the finished product both as a biologic and as a nanomaterial, requiring parallel advancement of CMC, non‑clinical, and ethics packages. This is increasingly supported by alignment across jurisdictions – through Europe's draft guideline on mRNA medicines (EMA/CHMP/BWP/82416/2025) and the FDA's guidance on drug products containing nanomaterials (FDA‐2017‐D‐0759). Other regulators, including the Pharmaceuticals and Medical Devices Agency, Japan and Therapeutic Goods Administration, Australia, are also beginning to adapt their frameworks for advanced modalities, reflecting a broader trend towards global alignment. In the United States, the FDA has introduced a Platform Technology Designation Program. Sponsors whose manufacturing platform has already supported an approved product can request this designation, enabling them to leverage prior CMC and, where applicable, non‑clinical data when filing for new indications, thereby streamlining development and review (see FDA's draft guidance on the Platform Technology Designation Program). The mechanism is conditional, and the FDA can withdraw the status if emerging safety signals show that platform‐level assumptions no longer hold, but it signals the agency's intent to reward well‐characterized, reusable mRNA‐LNP infrastructures and could markedly shorten the regulatory runway for future brain‐directed therapeutic candidates.

Because an LNP is regulated as a combination product, sponsors map each material attribute to an orthogonal release assay and acceptance limit, an approach that regulators have started to endorse (e.g., the FDA's and EMA's guidelines stated above). First‐in‐human trials are expected to open with ultra‐rare, monogenic neurodegenerative disorders (where benefit–risk is clearest) using adaptive dose‐escalation designs that mix intrathecal and intravenous cohorts and have translational surrogate endpoints to guide dosing in clinical trials instead of just relying on long‐term clinical endpoints.

Alongside the technical dossier, early engagement with ethics committees, neurosurgeons, and patient‐advocacy groups is essential, particularly for cognitively vulnerable populations such as AD. This is especially important given the certain level of stigma associated with mRNA therapy. Clear consent language must explain that mRNA expression is transient, repeat dosing is probable, and intrathecal delivery may be involved. Public familiarity with mRNA vaccines can ease conversations with ethics committees and insurers, yet public opinion is not uniformly positive: pockets of vaccine hesitancy, often driven by misinformation, persist and could slow recruitment if therapeutic trials are conflated with immunization campaigns. Proactive education that distinguishes transient, non‐integrating mRNA therapeutics from vaccines, combined with transparent safety data, will therefore be essential before outcome‐linked reimbursement models piloted in metabolic disorders can translate to neurodegenerative indications. Taken together, converging regulatory guidance, standardized CMC frameworks, and proactive stakeholder strategies outline a credible path from proof‐of‐concept to bedside for BBB‐shuttling mRNA‐LNPs.

## 8 Challenges and Knowledge Gaps

**FIGURE 5:**
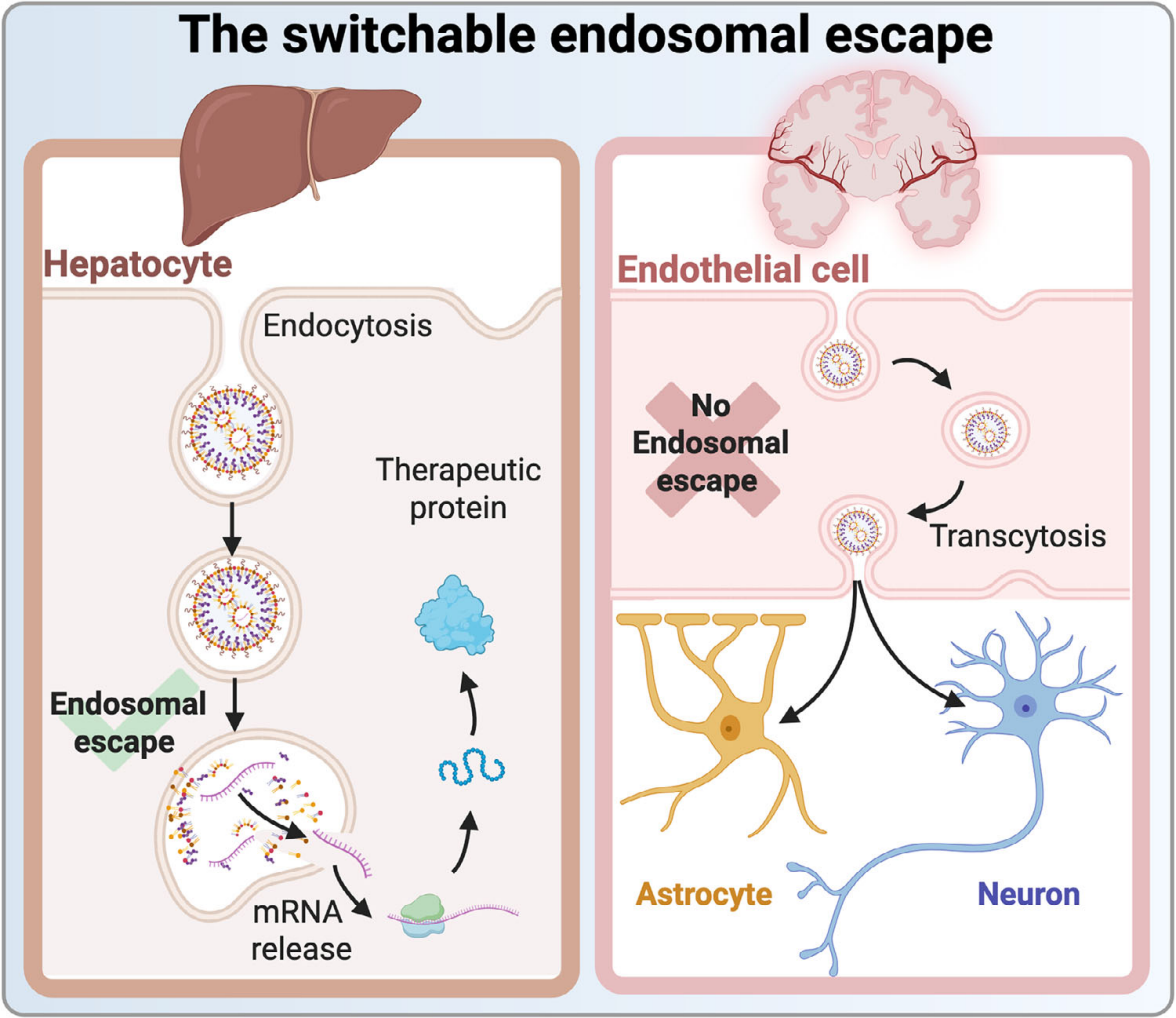
The switchable escape dilemma in CNS‐directed mRNA‐LNP delivery. mRNA‐LNP delivery to peripheral tissues benefits from enhanced endosomal escape within target cells, enabling efficient cytosolic release of mRNA and high therapeutic protein production (left). In contrast, brain‐targeted mRNA‐LNPs first encounter BBB endothelial cells, where endosomal escape is undesirable: premature release leads to luminal or endothelial protein deposition and depletes the therapeutic dose before it can reach the brain parenchyma (right). After transcytosis or barrier passage, however, the same LNP must then efficiently escape endosomes within neurons, astrocytes, or microglia to ensure productive cytosolic delivery. CNS‐directed LNPs therefore require context‐dependent, compartment‐specific control of endosomal escape, a design challenge not encountered in liver‐ or tumour‐targeted LNP platforms.

Rapid progress aside, several bottlenecks still stand between convincing proof‐of‐concept rodent results and real clinical benefit. The most immediate is delivery: systemic LNPs must first traverse an intact BBB and then release mRNA only after they have crossed it. That dual mandate creates a uniquely brain‐specific “switchable escape” dilemma (Figure 5). Endosomal escape is undesirable in BBB endothelial cells because premature release strands the translated protein on the luminal or endothelial surface and depletes the dose before it enters the parenchyma. Yet endosomal escape becomes indispensable once the same particle is internalized by neurons, astrocytes, or microglia, where cytosolic release is required for therapeutic protein synthesis. Conventional ionizable lipids optimized for liver or tumour targets don't face this compartment‐specific constraint and therefore lack the selective control needed for CNS applications. Addressing this challenge will require chemistries or cofactors that suppress escape in the endothelium while enabling robust escape in brain‐resident cells. Ageing introduces an additional layer of complexity because BBB remodelling is heterogeneous across regions, meaning that no single lipid p*K*a or surface architecture is likely to be optimal for all parts of the aged human brain. Standard efficacy screens in three‐month‐old mice often overpredict uptake in older adults, but emerging microfluidic BBB‐on‐a‐chip models seeded with senescent human endothelial cells and pericytes are beginning to bridge this translational gap [188, 189, 190].

The immunology of chronic dosing poses yet another hurdle. PEGylated shells risk accelerated blood clearance as anti‐PEG IgM/IgG titers rise and complement pathways activate; stealth strategies include masked PEG linkers that expose the hydrophilic chain only after endosomal acidification and zwitterionic polymers such as PMPC that evade preformed antibodies entirely [191, 192, 193]. Fast‐clearing, biodegradable ionizable lipids offer an immunological advantage: because the lipid components themselves are broken down and eliminated within a few days, the innate immune system is not continually exposed to the same antigenic surface, reducing the risk of anti‐lipid or anti‐PEG antibody build‐up between doses. Recent work on the next generation of ionizable lipids has focused on incorporating biodegradable linkers, such as ester or carbonate moieties, to shorten lipid persistence in tissues and thereby reduce the likelihood of cumulative immune activation during repeated dosing [194]. Although these studies have so far been performed primarily in systemic or hepatic mRNA‐LNP applications, the underlying principles are directly relevant for CNS‐directed formulations, where chronic administration is likely to be required. In parallel, alternatives to PEGylation, including zwitterionic and polysarcosine‐based surface chemistries, have been proposed to mitigate complement activation and the formation of anti‐PEG antibodies observed with PEG‐containing therapeutics [195, 196]. While data in mRNA‐LNP systems remain limited, these emerging strategies may offer future options for improving tolerability. Additionally, immune tolerization approaches such as optimized dose‐escalation schedules, reduced initial priming doses, or co‐formulation with agents that blunt acute innate responses have been reported in systemic mRNA‐LNP contexts and highlight the importance of proactive immunogenicity management as CNS‐targeted mRNA therapeutics move towards multi‐dose regimens.

On the CMC front, next‐generation lipids often require three‐ to five‐step esterification with moisture‐sensitive intermediates, a recipe for low throughput at large scale; however, continuous‐flow chemistry combined with AI‐guided retrosynthetic planning now allows chemists to identify scalable routes more quickly and optimize multi‐step lipid syntheses in less time than traditional trial‐and‐error methods. Finally, reproducibility is likely to remain a weak spot unless reporting practices improve. Variations in how studies normalize dose, distinguish mRNA from protein endpoints, or present immunogenicity profiles make it hard to compare formulations across laboratories. One way forward would be to agree on a “Minimum Information for Lipid‐Nanoparticle Experiments” (MI‐LNP) checklist (analogous to MIAME for microarrays) that encourages authors to share basic parameters plus complementary multi‐omics data (transcriptomics, lipidomics, cytokine panels) and raw biodistribution maps in an open repository similar to the Gene Expression Omnibus. Such a framework would give the community a common denominator for future meta‐analyses and machine learning models. Building on this idea, we propose that the MI‐LNP framework could substantially improve reproducibility and comparability across CNS‐directed mRNA‐LNP studies. Such a checklist would (i) include standardized reporting of core physicochemical parameters (particle size, polydispersity, zeta potential, lipid composition), (ii) dosing details and administration route, (iii) quantitative biodistribution and cell type‐resolved expression readouts, (iv) immune activation and tolerability assessments, and (v) access to underlying spatial or omics datasets when available. Consistent reporting of these elements would enable more rigorous cross‐study benchmarking, facilitate meta‐analyses, and accelerate rational lipid discovery, particularly important in the CNS field, where even small differences in BBB integrity, age, or neuroinflammatory state can markedly influence LNP performance.

## 9 Future Directions and Emerging Concepts

Innovation in mRNA‐LNP therapeutics is advancing on four mutually reinforcing fronts: materials science, payload design, manufacturing logistics, and AI optimization, and each promises to unlock new clinical territory. One of the most striking trends is the emergence of programmable, stimulus‐responsive particles: by grafting pH‐labile hydrazone bonds or reactive‐oxygen‐species (ROS)‐cleavable thioketals into the ionizable lipid, formulators can create LNPs that circulate inertly but jettison their PEG cloak or unload cargo only within inflamed, oxidative micro‐domains [197]. In a murine traumatic‐brain‐injury model, ROS‐responsive lipids increased the on‐target relative to off‐target protein expression compared with non‐responsive controls, demonstrating that precise spatiotemporal control is achievable [197]. Payload design is evolving just as quickly. Early proofs of concept showed that co‐encapsulating two distinct mRNAs (for example, a therapeutic enzyme plus a reporter) does not compromise colloidal stability and ensures that both transcripts follow the same biodistribution and clearance profile. Subsequent vaccine work has pushed the envelope to multiple mRNAs in a single LNP, demonstrating that multivalent formulations can be manufactured at scale and retain balanced expression of each component [198]. Those successes make it plausible to explore multi‐payload regimens for complex disorders such as AD, where simultaneous delivery of neurotrophic, anti‐inflammatory, and proteostasis‐modulating transcripts could be advantageous.

On the manufacturing front, containerized GMP suites are being deployed in several countries, allowing in‐region synthesis that trims cold‐chain mileage and even permits population‐specific lipid tuning, such as adjusting PEG density to local anti‐PEG‐antibody prevalence. Finally, AI‐driven formulation discovery is compressing the design–build–test–learn cycle: generative chemical models trained on large datasets of published lipids can propose novel scaffolds pre‐optimized for p*K*a, log*P*, and biodegradability within minutes, after which physics‐based simulations triage millions of virtual candidates [199].

Over the next few years, these converging advances are expected to yield the first clinical‐grade brain‐penetrant LNP, initiate Phase I trials of combination mRNA + small‐molecule regimens, and bring regulators the inaugural dossier for a fully containerized manufacturing facility milestones that would transform mRNA‐LNP therapeutics from a pandemic‐borne technology into a mature, versatile platform for neurodegenerative medicine.

## 10 Concluding Remarks

Over the past five years, lipid chemistry, RNA engineering, and large‐scale manufacturing have converged into a robust platform for systemic mRNA therapeutics, while geroscience has clarified how vascular ageing reshapes the BBB and innate immunity, turning those biological insights into concrete design targets for BBB‐shuttling nanoparticles. The intersection of these two knowledge streams – materials innovation on one side and ageing biology on the other – opens a unique window for CNS applications. Preclinical studies now show that rationally tuned LNPs can deliver pharmacologically relevant mRNA doses to neurons and glia in rodent models, and parallel clinical programmes in metabolic liver disorders confirm the safety, manufacturability, and redosing feasibility of the underlying chassis. Regulators have taken note, codifying platform‐based review pathways that let new payloads advance under the umbrella of established critical quality attributes.

The remaining obstacles are demanding yet fairly well‐delineated. Endosomal escape per se is already adequate for liver‐directed products; the challenge for brain applications is balancing that escape efficiency with the need for particles to cross the BBB intact. Lipid libraries will therefore have to couple BBB passage with “just‐enough” endosomal release in neurons and glia. Added to that, the patchy biology of the ageing barrier argues for region‐specific screening, and repeat‐dose regimens will still need better stealth chemistries or immune‐tolerizing strategies to avoid anti‐lipid and anti‐PEG responses over time. Each challenge already has first‐generation solutions such as fusogenic proteolipids, region‐aware lipid screens, and masked or zwitterionic stealth polymers, suggesting no fundamental barrier stands in the way. If current trajectories hold, a Phase I trial of an mRNA‐LNP therapy for a rare monogenic neurodegenerative disease could begin within three to five years, paving the way for broader AD or PD studies later in the decade. Success would herald a programmable and scalable therapeutic class capable of addressing proteinopathies, gene deficits, and even epigenetic dysregulation with unprecedented agility.

The future will require cross‐disciplinary consortia that blend ageing neuroscience, AI‐assisted mRNA design, programmable lipid chemistries, and patient‐specific therapeutic strategies, which may enable personalized mRNA medicines tailored to genetic and molecular disease profiles. However, key challenges remain, including achieving cell‐type‐specific delivery in heterogeneous human brain tissue, managing chronic dosing immunogenicity, and ensuring scalable manufacturing of increasingly complex lipid formulations. With coordinated effort, the field can transform CNS drug development from a protracted, molecule‐by‐molecule slog into a nimble, code‐driven enterprise, meeting the urgent societal imperative to blunt the emerging dementia epidemic.

## Author Contributions

AAB reviewed the literature and wrote the manuscript. DF contributed to writing and editing of the manuscript. FA prepared the figures and edited the manuscript. RN, SA, and AIB edited the manuscript. All authors have read and approved the final version of the manuscript.

## Conflicts of Interest

AIB is a shareholder in Cogstate Ltd. and Alterity Ltd., has a profit share arrangement with Collaborative Medicinal Development Pty Ltd., and has received lecture fees from Biogen and NovoNordisk. AIB is a member of the *Exploration* editorial board, and he was not involved in the handling or peer review process of this manuscript. DLF is a shareholder in Zitra Medicines Pty Ltd. and Messenger Bio Pty Ltd.

## Data Availability

Data sharing not applicable to this article as no datasets were generated or analysed during the current study.
